# Noninvasive Neuromodulation Therapies: The Cure For Depression?

**DOI:** 10.1192/j.eurpsy.2025.886

**Published:** 2025-08-26

**Authors:** R. P. L. Andrade, V. H. Santos, N. Castro, I. Santos, F. Cunha, E. Almeida, J. Abreu, H. Afonso

**Affiliations:** 1 ULS Viseu Dão-Lafões, Viseu; 2 ULS Cova da Beira, Covilhã, Portugal

## Abstract

**Introduction:**

Depressive disorder is a common mental disorder, with an estimated 3.8% of the population experiencing it. Despite the advent of new antidepressant medication, many patients presenting with Major Depressive Disorder (MDD) do not recover after multiple trials. Although the prevalence of treatment-resistant depression (TRD) is not clear due to the lack of a standard definition, its prevalence ranges from approximately 30 to 70 percent.

**Objectives:**

Considering the high prevalence of treatment-resistant depression, this work aims to evaluate the effectiveness of alternative treatments, namely Noninvasive Neuromodulation Therapies in the treatment of MDD.

**Methods:**

Non-systematic literature review, using Pubmed as database, with the keywords “depression treatment”, “neuromodulation” and “noninvasive neuromodulation”.

**Results:**

We can divide non-invasive neuromodulation into convulsive therapies (CV) and therapies that do not involve inducing a seizure. Additionally, we can also divide them into clinically available therapies and others only available in investigational settings.

Regarding clinically available CV, we have Electroconvulsive Therapy (ECT), the oldest neurostimulation procedure. Being heavily studied, ECT is superior to pharmacotherapy for MDD based upon meta-analyses of randomized trials and is generally considered the most efficacious treatment for depression, albeit recurrence following remission is common.

Other CV, but still in investigational stages, are Magnetic seizure therapy (MST) and Focal electrically administered seizure therapy (FEAST) both showing positive results in prospective studies and MST in a small head-to-head randomized trials with ECT, that showed a similar efficacy between these two therapies.

Other clinically available, but not convulsive therapies, are Repetitive Transcranial Magnetic Stimulation (rTMS) and Cranial Electrical Stimulation (CES). Meta-analyses of randomized trials indicate that rTMS is beneficial for treating TRD, being also approved by the FDA. In its turn, multiple reviews indicate that no high-quality studies have demonstrated that CES is efficacious for MDD or TRD.

Additional non-convulsive therapies, available in investigational settings, include Transcranial Direct Current Stimulation, Transcranial Low Voltage Pulsed Electromagnetic Fields, Trigeminal Nerve Stimulation, Low Field Magnetic Stimulation and Transcutaneous Vagus Nerve Stimulation, with all of them showing positive effects in the treatment of MDD or TRD, except for Low field magnetic stimulation.

**Conclusions:**

With this review, we were able to verify that clinically available non-invasive neurodomulation therapies, such as ECT and rTMS, present robust results in the treatment of MDD and TRD, however, resistance to these therapies also exists.

Considering the positive results of multiple novelty therapies, these could be the solution to this scourge.

**Disclosure of Interest:**

None Declared

